# The Book World of Medicine and Science

**Published:** 1893-05-13

**Authors:** 


### THE BOOK WORLD OF MEDICINE AND SCIENCE.

### BOOK REVIEWS.

FROM HOSPITAL WARD TO CONSULTING ROOM, WITH NOTES BY THE WAY: A MEDICAL AUTOBIOGRAPHY. By a GRADUATE of the London University. (London: H. K. Lewis, 1893.)

Faithful autobiographies are valuable and interesting, but few people have the power of writing them. Instead of a simple record of the individual's life, they are apt to be a record of his experiences—a very different thing; anecdotes of the persons he has met, and dissertations upon all manner of relevant and irrelevant subjects. The autobiography before us is not of this class; it is very brief, limited to the simple statement of the writer's struggle to obtain a high professional position. Collateral subjects have been carefully eschewed, and we see only an honest man fighting the battle of life. Beginning life as the son of a tradesman in a Northern town, he was early bereft of both parents, and first at a dame's school and then at a grammar school, received a very defective education—a mere smattering of Latin and English. Before he was thirteen he was apprenticed to a relative who was in general practice, and he worked on here for some years, then he went as outdoor dispensing assistant to a general practitioner in Yorkshire for two years, and subsequently in Kent, his salary being about £1 per week. Thence he went as physician's assistant to a dispensary in Leeds, where, in addition to his regular work, he also attended lectures in the famous medical school. After a year of this work his health broke down, and he went into business as a chemist, and continued to earn an honourable livelihood by this means for many years. A chance remark of a friend served to rekindle his former ambition, and disposing of his business, he completed his medical education and obtained the diploma of the Apothecaries' Hall, and then that of the College of Surgeons. He then embarked in practice, but, being honourably ambitious, he determined to obtain the degree of M.D. of the University of London. Hard at work in general practice, far away from London and any medical school, the preparation for the University examinations was difficult, but, in spite of failures, he plodded on, and in due time was awarded the much-prized degree. This step gained, he determined to improve his practice, and become a consultant; and, although at first he was thwarted by the jealousy and indifference of fellow-practitioners and by bad health, he ultimately succeeded in his object. The main lesson of this story is a very old and simple one—the reward of perseverance. It shows a man of no great ability, without early advantages and handicapped by indifferent health, attaining to a high position in his profession by setting before himself a high and worthy standard and allowing no difficulty, no obstacle, to stand in the way of his attaining it. It is an old lesson, but not too old to tell again, and one that is found ever new and great by those who learn it by their own industry.

### THE MAY REVIEWS.

PROFESSOR MAX MULLER gives an amusing exposure of Madame Blavatsky in the NINETEENTH CENTURY in his article on "Esoteric Buddhism." "To quote her blunders," he says, "would be endless. Of what character they are will be seen when I quote what she says about the serpent being

**The Hospital Advertiser.**

THE BOOK WORLD OF MEDICINE AND SCIENCE.—(*continued*).

the good or the evil spirit. 'In this case,' she writes, 'the serpent is the Agathodaimon, the good spirit; in its opposite aspect, it is the Kakothodaimon, the bad one.' I believe that this mistake, when I pointed it out to an undergraduate friend of mine at Oxford, saved him from enrolling himself as an Esoteric Buddhist."

An article in the New Review on "The Propagation and Prevention of Cholera," by Dr. Roose, traces the history of the cholera with the successive theories which have been held with regard to its spread. Under the head of precautions he has nothing new to urge, but perhaps the old cautions cannot be reiterated too loud or too often.

Mr. Routh in the National Review uplifts "The Bitter Cry of the Superannuated." As regard the profession of teaching there is no room to doubt the serious hardship inflicted by the preference for younger (and cheaper) labour. But is there not a little exaggeration in the lament of Mr. Routh on behalf of the parson and the doctor? Do the majority of patients really persist in showing "a thoughtless preference for none but the most youthful medical men, irrespectively of other qualifications"?

In the Westminster Review a paper by Mr. Bulman, "Are Bacilli Causes of Disease?" embodies his conviction that the case in point has not been proved, and must therefore be re-tried. It appears there are still sceptics who would treat cholera as a non-contagious, nervous disorder, and who regard the cholera bacillus as "a necessary aid to digestion." To these the article will commend itself.

---

Among the publishers' announcements of interest to our readers we notice the following:—

A new edition of "The Pocket Gray," by E. Cotterell, being a popular *vade mecum* with students in their second year.

The fourth edition of Sir W. Dalby's "Diseases and Injuries of the Ear."

The fourth edition of Dr. Dalles' "Accidents and Emergencies."

A pamphlet on "Recreation," by W. Odell, giving information from headmasters of several public schools.

Part 2 of R. B. Sharpe's beautiful monograph on "Birds of Paradise and Bower Birds."

S. R. Bottone has written a popular introduction to "The Study of Electricity and Magnetism," and from the pen of A. K. J. Browne we have an "Elementary Handbook of Geology."

Smith and Elder announce a text-book of "Vertebrate Embryology," by Professor A. M. Marshall, which is sure to be acceptable to all students of this most important and interesting branch of biology. The same firm announce the eighth edition of Playfair's treatise on "Midwifery."

Longmans, Green, and Co. are about to publish a posthumous work on the "Glacial Geology of Great Britain and Ireland," compiled by Dr. Crosskey from the papers of the late Henry Carvill Lewis.

G. Reid has written a work on "Practical Sanitation," which contains a chapter on sanitary law by Mr. H. Manby.

Messrs. Swan Sonnenschein and Co. will shortly publish a work by Dr. Berdoe, of Bristol, entitled "The Healing Art: a Popular History of the Origin and Growth of Medicine in all Ages and Countries." Dr. Berdoe is an acknowledged authority on all anthropological questions, and this work will contain chapters on the history of medicine from this point of view. The work is sure to be of great importance and full of interest.

